# Two Classes of Bacterial IMPDHs according to Their Quaternary Structures and Catalytic Properties

**DOI:** 10.1371/journal.pone.0116578

**Published:** 2015-02-23

**Authors:** Thomas Alexandre, Bertrand Rayna, Hélène Munier-Lehmann

**Affiliations:** 1 Institut Pasteur, Unité de Chimie et Biocatalyse, Département de Biologie Structurale et Chimie, 28 rue du Dr Roux, F-75015, Paris, France; 2 Centre Nationale de la Recherche Scientifique, Unité Mixte de Recherche 3523, F-75015, Paris, France; 3 Université Paris Diderot, Sorbonne Paris Cité, F-75205, Paris, France; 4 Institut Pasteur, Proteopole, Plateforme de biophysique des macromolecules et de leurs interactions, 25 rue du Dr Roux, F-75015, Paris, France; 5 Centre Nationale de la Recherche Scientifique, Unité Mixte de Recherche 3528, F-75015, Paris, France; University of Queensland, AUSTRALIA

## Abstract

Inosine-5′-monophosphate dehydrogenase (IMPDH) occupies a key position in purine nucleotide metabolism. In this study, we have performed the biochemical and physico-chemical characterization of eight bacterial IMPDHs, among which six were totally unexplored. This study led to a classification of bacterial IMPDHs according to the regulation of their catalytic properties and their quaternary structures. Class I IMPDHs are cooperative enzymes for IMP, which are activated by MgATP and are octameric in all tested conditions. On the other hand, class II IMPDHs behave as Michaelis-Menten enzymes for both substrates and are tetramers in their apo state or in the presence of IMP, which are shifted to octamers in the presence of NAD or MgATP. Our work provides new insights into the IMPDH functional regulation and a model for the quaternary structure modulation is proposed.

## Introduction

Allosteric regulation [1], where the binding of an effector at one site can induce structural changes that are transmitted to distal active site(s), is one of the various strategies to control activity of proteins. Cross-talk communication between subunits enables fine tunings of activity in response to slight changes in substrate concentration. This mechanism has been largely documented (two special issues in 2013 [2,3]) and a colloquium (“Allosteric Interactions in Cell Signaling and regulation”) was recently dedicated to allostery to mark the 50^th^ anniversary of its discovery. Besides the classic models (concerted model of Monod, Wyman and Changeux [4] and sequential model of Koshland, Nementhy and Filmer [5]), a novel concept for allosteric regulation has emerged more recently with morpheeins [6]. This model implies the existence of distinct quaternary structure assemblies of the same protein. The dynamic equilibrium between the various multimers can be shifted by the binding of a ligand, either by blocking or favoring the formation of one of the multimers. Porphobilinogen synthase constitutes the morpheein prototype [7], and an increasing number of proteins exhibits characteristics consistent with this model [8]. Cystathionine-ß-synthase presents also characteristics consistent with the morpheein model [6]. This protein contains CBS modules (these modules were first identified in the cystathionine-ß-synthase), which might play a pivotal role in this allosteric regulation. Other proteins such as inosine-5′-monophosphate dehydrogenase (IMPDH) contains these regulatory modules, whose function might be controlled by this mechanism.

IMPDH (E.C. 1.1.1.205) is widely distributed in nature and occupies a key position in purine nucleotide metabolism [9,10]. This enzyme catalyzes the rate-limiting NAD-dependent oxidation of IMP to XMP. The work of Kozhevnikova *et al*. [11] has recently revealed an additional function in drosophila, where IMPDH was reported to be a DNA-binding transcriptional repressor.

IMPDH shares a two-domain organization composed of one catalytic domain, a (ß/α)_8_ barrel, and a smaller flanking domain, containing two CBS modules [12–14] (forming together the so-called Bateman domain [15]). We have recently identified a bacterial IMPDH (*Pseudomonas aeruginosa* IMPDH (IMPDHpa)), which is a cooperative enzyme for IMP [16]. MgATP was found to be a positive effector of IMPDHpa acting on the maximal rate and on the affinity for IMP. This positive effector binds onto the two CBS modules, with consequences on the global shape as evidenced by our studies on IMPDHpa and on the human IMPDH1 [16]. Indeed, the octameric macromolecular organization, that has been overlooked up to now, is significantly changed upon addition of MgATP, especially in the case of the human IMPDH1, where the two observed octameric species can stack up into isolated fibres.

In the present study, we have chosen bacterial IMPDHs from different human pathogens. Out of the eight recombinant enzymes expressed and purified, seven were further characterized by a combination of biochemical and biophysical approaches. We have focused our interest on the regulation of the quaternary structure and of the catalytic activity. We have identified two classes of bacterial IMPDHs: class I with an octameric organization and cooperative kinetics, and class II being either tetrameric or octameric, and exhibiting michaelian kinetics.

## Materials and Methods

### Chemicals, enzymes and bacterial strains

*Bacillus thuringiensis* serovar monterrey BGSC 4AJ1 strain was provided by Pr Anne-Brit Kolstø. Genomic DNAs from *Acinetobacter baumannii* strain 5377 (ATCC 17978), *Burkholderia thailandensis* strain E264 (ATCC 700388), *Legionella pneumophila* subsp. *pneumophila* strain Philadelphia-1 (ATCC 33152D-5) and *Neisseria meningitidis* strain FAM18 (ATCC 700532) were purchased from ATCC, whereas genomic DNAs from *Klebsiella pneumonia* strain 52145, *Staphylococcus aureus* strain N315 and *Bacillus thuringiensis* serovar monterrey BGSC 4AJ1 were kindly provided by Dr R. Tournebize, Dr M. Débarbouillé and Dr P. Goossens, respectively. DNA polymerases, restriction enzymes, T_4_ DNA ligase were from New England Biolabs. Oligonucleotides were purchased from Eurofins MWG Operon. BD TALON metal affinity resin was from BD Biosciences Clontech. Nucleotides, nicotinamide adenine dinucleotide (NAD) and 1-anilino-8-naphtalene sulfonate (ANS) were from Sigma. Adenosine-5′-triphosphate-γ-(sulfo-1-naphtyl)amide, triethylammonium salt (ATP-γ-AmNS) was purchased from Jena Bioscience.

### Plasmids and growth conditions

*guaB* genes from different bacteria (*A*. *baumannii*, *B*. *thailandensis*, *B*. *thuringiensis* serovar monterrey BGSC 4AJ1, *K*. *pneumoniae*, *L*. *pneumophila* subsp. *pneumophila*, *N*. *meningitidis*, and *S*. *aureus*) were amplified by PCR from genomic DNA as template using the corresponding primers (Table 1). They were cloned into a pET28a plasmid (Novagen, Inc.) between the NdeI and HindIII (except for the PCR product from *B*. *thuringiensis* serovar monterrey BGSC 4AJ1, where HindIII was replaced by XhoI) restriction sites. The resulting plasmids (Table 1) were introduced into the *E*. *coli* strain BL21(DE3)/pDIA17 [17] to overproduce the different IMPDHs. All plasmids were sequenced to verify their identity (see acronyms in Table 1). Plasmid pHL143–1 for IMPDHpa expression has been described previously [16]. Bacteria were grown at 37°C in 2YT medium supplemented with 35 μg/ml kanamycin and 30 μg/ml chloramphenicol. Production of recombinant proteins was induced with 1 mM IPTG when cultures reached an absorbance of 1.5 at 600 nm. Bacteria were harvested by centrifugation 4h after induction.

**Table 1:** Primers used for cloning the *guaB* gene from various bacteria and names of the corresponding plasmids.

Organism	Plasmid name	Flanking primers
*A*. *baumannii*	pHL160–2	Forward 5′-GGAATTCCATATGCTGACCATCGTTCAAGAA-3′
		Reverse 5′-CCCCAAGCTTCTAACCAACACGATAGTTCGGA-3′
*B*. *thailandensis*	pHL161–1	Forward 5′-GGAATTCCATATGCGTCTGATCCAAAAAGCAC-3′
		Reverse 5′-CCCCAAGCTTTCAGTCCACGTGATAGTTGGG-3′
*B*. *thuringiensis*	pHL165–1	Forward 5′-GGAATTCCATATGTGGGAATCTAAATTTGTTTAAAGAAGGTCTGACTTTT-3′
serovar monterrey BGSC 4AJ1		Reverse 5′-CCGGCTCGAGTTATAATGAGTAGTTTGGAGCCTCTTTTGTAATTTGTAC-3′
*K*. *pneumoniae*	pHL162–2	Forward 5′-GGAATTCCATATGCTACGTATCGCTAAAGAAG-3′
		Reverse 5′-CCCCAAGCTTTCAGGAGCCCAGACGGTAG-3′
*L*. *pneumophila* subsp. *pneumophila*	pHL163–1	Forward 5′-GGAATTCCATATGCCTCTATCCATTGTGCAACA-3′
		Reverse 5′-CCCCAAGCTTTTAATTATCCACCTGATAATTGGGG-3′
*N*. *meningitidis*	pHL164–1	Forward 5′-GGAATTCCATATGCGTATCGTAGAAAAAGCCTATAC-3′
		Reverse 5′-CCCCAAGCTTTCAGCGGTGGTAGTTCGGTG-3′
*S*. *aureus*	pHL146–2	Forward 5′-GGAATTCCATATGTGGGAAAGTAAATTTGCAAAAGAATCATTAACGTTTG-3′
		Reverse 5′-CCCCAAGCTTTTAGAATGAGTAGTTCGGTGATTCTTTCGTAATTTG-3′

### Purification of IMPDHs

Bacterial pellet expressing recombinant IMPDH was resuspended in buffer A (see Table 2) supplemented with Complete Protease Inhibitor Cocktail EDTA-free (Roche) and disrupted by sonication. After centrifugation at 15,000 g for 45 minutes, the supernatant was applied to BD TALON resin using the batch/gravity-flow column purification procedure at room temperature [18]. Washing steps were performed using the corresponding buffer A and 10 mM imidazole, and the protein was eluted with buffer A and 150 mM imidazole (except for the *A*. *baumannii* enzyme where 250 mM imidazole was used). Fractions containing the protein were immediately dialyzed against buffer A (with 2 mM DTT or 1 mM TCEP for IMPDHba) using a Spectra/Por membrane with a molecular weight cut off of 12–14 kDa and when necessary, further concentrated (up to 10 mg/ml) using a 50K stirred cell system (Pall Life Sciences). The purified proteins were stored at 4°C.

**Table 2:** Composition of buffers used for purification and enzymatic assay (buffer A and reaction medium, respectively) for each IMPDH (named by an acronym).

Organism	Acronym	Buffer A	Reaction medium
*A*. *baumannii*	IMPDHab	K_2_HPO_4_ 50 mM pH8.5 KCl 100 mM	K_2_HPO_4_ 50 mM pH8 KCl 100 mM
*B*. *anthracis*	IMPDHba	Tris-HCl 20 mM pH8.5 KCl 20 mM	Tris-HCl 50 mM pH9 KCl 150 mM
*B*. *thailandensis*	IMPDHbt	Na_2_CO_3_ 50 mM pH9.5 KCl 100 mM	K_2_HPO_4_ 50mM pH8 KCl 100mM
*K*. *pneumoniae*	IMPDHkp	K_2_HPO_4_ 50mM pH7.5 KCl 100mM	K_2_HPO_4_ 50mM pH8.5 KCl 100mM
*L*. *pneumophila* subsp. *pneumophila*	IMPDHlpp	K_2_HPO_4_ 50mM pH9 KCl 50mM	Na_2_CO_3_ 50mM pH10 KCl 20mM
*N*. *meningitidis*	IMPDHnm	K_2_HPO_4_ 50mM pH9 KCl 20mM	K_2_HPO_4_ 50mM pH8 KCl 100mM
*P*. *aeruginosa*	IMPDHpa	K_2_HPO_4_ 50mM pH8 KCl 100mM	Tris-HCl 50mM pH8 KCl 100mM
*S*. *aureus*	IMPDHsa	K_2_HPO_4_ 50mM pH8 KCl 50mM	Na_2_CO_3_ 50mM pH9.2 KCl 20mM

Size-exclusion chromatography was performed on a HiLoad 26/60 Superdex 200 prep grade (GE Healthcare) calibrated with protein standards (ferritin 440 kDa, pyruvate kinase 228 kDa and aldolase 158 kDa).

### Enzymatic assays and kinetic characterization

IMPDH activity was determined at 30°C by monitoring the formation of NADH in 0.5 mL final volume on an Eppendorf ECOM 6122 photometer. The reaction buffer is detailed for each IMPDH in Table 2. Assays were performed at different concentrations of IMP, NAD, ATP, and MgCl_2_, to which IMPDH (final concentration range: 0.18–1.8 μM) diluted in buffer A (see Table 2) was added to start the reaction. One unit of enzyme activity corresponds to 1 μmole of the product formed in 1 min. Experimental data were fitted using the Kaleidagraph software according to the Michaelis-Menten equation v = V_m_ [S]/(K_m_ + [S]), the substrate inhibition equation v = V_m_ [S]/(K_m_ + [S] + [S]^2^/K_I_), or to the Hill equation v = V_m_ [S]^nH^ /(K_0.5_^nH^ + [S]^nH^), where v is the reaction rate, V_m_ the maximal rate, [S] the NAD or IMP concentration, K_m_ the Michaelis-Menten constant, K_I_ the inhibitory constant, K_0.5_ the IMP concentration at half-saturation, and n_H_ the Hill number index.

### Circular dichroism

CD spectra were measured on an Aviv 215 CD spectrometer (Aviv Biomedical) with protein samples at 1 mg.mL^−1^ in the corresponding buffer A (see Table 2).

Far-UV spectra were recorded at 20°C between 190 and 260 nm using a cylindrical quartz cell with 0.02 cm path length. Scans were repeated consecutively three times and merged to produce an averaged spectrum. It was further corrected using buffer baselines measured under the same conditions and normalized to the molar peptide bond concentration and path length as mean molar differential coefficient per residue. Secondary structure estimations were derived from the normalized spectra using the CDSSTR method included in the CD Pro software.

### Analytical ultracentrifugation

Sedimentation velocity experiments were performed at 20°C in a Proteomelab XL-I analytical ultracentrifuge (Beckman Coulter) equipped with double-UV and Rayleigh interference detection. Samples were prepared in their respective buffer A and spun at 42,000 rpm using an An-60 Ti rotor and 3 or 12 mm double sector Epon centerpieces. 10 mM IMP, 3 mM NAD or 3 mM MgATP were added to the corresponding buffer A for experiments in the presence of the substrates or the effector. The partial specific volumes of IMPDHs (0.738 ml/g for each, but for IMPDHba 0,742ml/g, IMPDHnm 0,739ml/g and IMPDHsa 0,741ml/g) were estimated from their amino acid sequences using the software Sednterp 1.09 (available online from The Boston Biomedical Research Institute). The same software was used to estimate buffer density (ρ = 0.9998, 1.0108, 1.0103, 1.008, 1.006, and 1.008 g/ml for IMPDHba, IMPDHbt, IMPDHkp IMPDHlpp, IMPDHnm, and IMPDHsa, respectively) and viscosity (η = 1.007, 1.0203, 1.0190, 1.0198, 1.0202 and 1.0257 cP for IMPDHba, IMPDHbt, IMPDHkp, IMPDHlpp, IMPDHnm and IMPDHsa, respectively). Absorbance and interference profiles were recorded every 5 min. Sedimentation coefficient distributions c(s) were determined using the software Sedfit 14.1 [19,20]. All the sedimentation coefficients are expressed in standard conditions (20°C, water).

### Fluorescence spectroscopy

Measurements were performed on a FP-6200 spectrofluorimeter (Jasco) in a Peltier-thermostated cell holder, using a 3 mm path length quartz cell (105.251 QS, Hellma). A bandwidth of 5 nm was used for the excitation and emission beams. For ATP-γ-AmNS fluorescence, the excitation wavelength was fixed at 323 nm and the emission spectra were recorded at 30°C, from 300 to 400 nm at a scan rate of 125 nm/min. The ATP-γ-AmNS fluorescent concentration was 1 μM in buffer A (see Table 1) supplemented with 5 mM MgCl_2_. The protein was progressively added in the cuvette (at final concentrations ranging from 0 to 190 μM). The ratio of fluorescence intensity at 420 nm to 500 nm (FIR 420/500) was used to follow the binding of ATP-γ-AmNS to the protein. For each protein concentration, the average of three values obtained from emission spectra that were corrected for blank measurements was considered. For ANS fluorescence, the emission spectra were recorded at 30°C, from 460 to 540 nm (excitation at 360 nm) at a scan rate of 125 nm/min. The fluorescence intensity was measured at 495 nm.

### Other analytical procedures

Protein concentration was measured according to Bradford [21], using a Bio-Rad kit or by amino acid analysis on a Beckman system 6300 high-performance analyser after 6 N HCl hydrolysis for 22 h at 110°C. SDS-PAGE was performed as described by Laemmli [22]. Dynamic light scattering (DLS) experiments were performed on a Dynapro plate reader (Wyatt technology).

## Results

### Overproduction, purification and quaternary structure characterization

**Fig 1:**
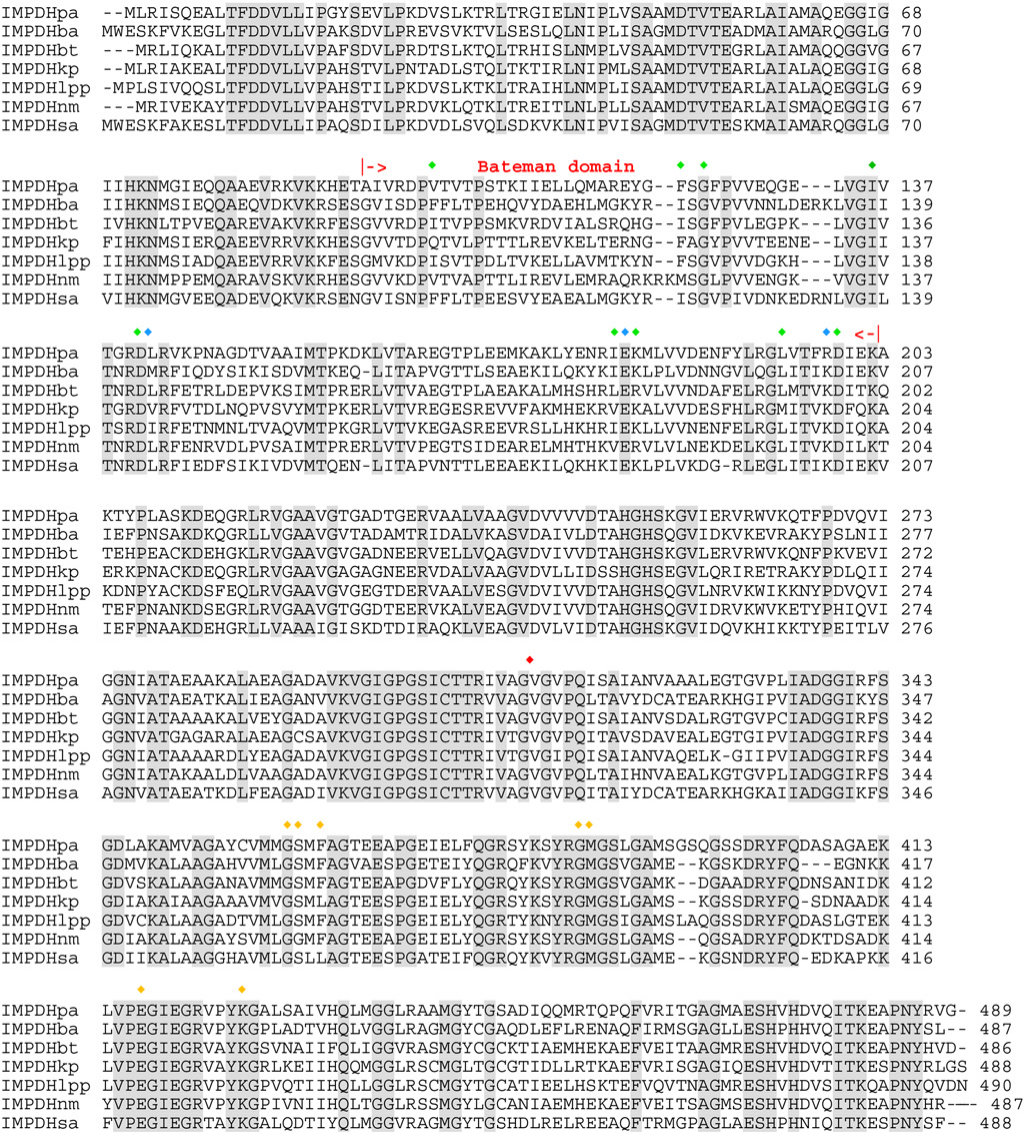
Primary sequence alignment of eight bacterial IMPDHs given by their acronym and explored in this work. Identical residues are shaded. CBS modules of IMPDHpa are marked out by arrows. IMPDHpa residues involved in the interaction with ATP, Mg and IMP are marked out by red, green and yellow diamonds, respectively. The catalytic cysteine residue is marked out by a red diamond. ba: *Bacillus anthracis*; bt: *Burkholderia thailandensis;* kp: *Klebsiella pneumonia*; lpp: *Legionella pneumophila* subsp. *pneumophila*; nm: *Neisseria meningitidis*; pa: *Pseudomonas aeruginosa*; sa: *Staphylococcus aureus*.

*guaB* genes of *A*. *baumannii*, *B*. *thailandensis*, *K*. *pneumonia*, *L*. *pneumophila*, *N*. *meningitidis* and *S*. *aureus* were annotated as loci A1S_3321, BTH_I2056, KPK_1283, lpg1723, NMC1103 and SA0375, respectively. In the case of *A*. *baumannii guaB* gene, the deduced primary sequence was 48 amino acids shorter in the N-terminal part than that of the other IMPDHs (Fig. 1). A careful inspection of the upstream region of the locus A1S_3321 has revealed another putative start codon, leading to a 144 bp extension of the coding sequence. For safety reason, *guaB* gene from *B*. *thuringiensis* serovar monterrey BGSC 4AJ1 was used as a template as the corresponding IMPDH sequence was 100% identical to that of *B*. *anthracis* IMPDH, which structural characterization was recently reported [23]. The different putative *guaB* genes were cloned into a pET vector as described into the Materials and Methods section.

The corresponding recombinant proteins were overexpressed in *E*. *coli* as soluble proteins and purified using affinity chromatography at over 95% purity as indicated by SDS-PAGE (data not shown). DLS experiments were performed to optimize purification and storage conditions (such as buffer composition, see Table 2) in order to increase the homogeneity and the stability of the recombinant protein samples. No satisfactory condition was found for IMPDHab, which was not further studied as it forms a turbid solution, regardless of the protein concentration or buffer composition (although a significant IMPDH catalytic activity was recorded). Analysis of circular dichroism spectra of the recombinant IMPDHs has revealed similar secondary structures with around 37% of alpha helix content, 17% of beta sheet and 12% of turn (data not shown).

**Fig 2:**
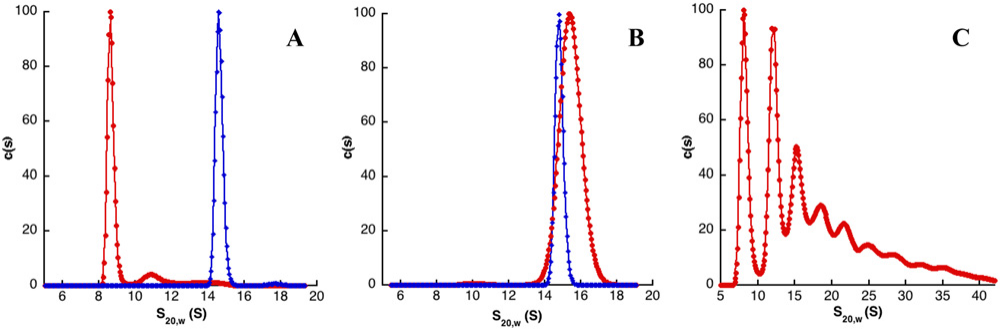
Continuous sedimentation coefficient distribution analysis of IMPDHbt (A), IMPDHlpp (B) and IMPDHkp (C) at 1 mg/mL (corresponding to 18.5, 18.3 and 18.4 μM, respectively) in the absence (red curves) or presence of 3 mM MgATP (blue curves). Sedimentation coefficients (S_20, w_) are expressed in Svedberg units (1S = 10^-13^s) and c(S) corresponds to normalized continuous size distribution.

The quaternary structure arrangement was first investigated by size exclusion chromatography. IMPDHbt, IMPDHlpp, IMPDHnm, IMPDHpa and IMPDHsa eluted in a single peak at the same volume as ferritin (440 kDa) for IMPDHlpp, IMPDHnm and IMPDHpa (compatible with the hydrodynamic size of an octamer) and as pyruvate kinase (218 kDa) for IMPDHbt and IMPDHsa (compatible with the hydrodynamic size of a tetramer). These results prompted us to further analyze by analytical ultracentrifugation experiments (performed at different IMPDH concentrations; Table 3 and Fig. 2) the oligomeric state and the hydrodynamic shape of the different IMPDHs selected in this study. We have also investigated the effect of the natural substrates (IMP and NAD) and MgATP: these experiments were done at a fixed IMPDH concentration (1 mg/mL) either in the absence (as a control) or in the presence of saturating concentrations of IMP, NAD, MgATP or a mixture of IMP and MgATP (Table 4). Two IMPDH classes can be distinguished from the results reported in Tables 3 and 4. In the first group composed of IMPDHlpp, IMPDHnm and IMPDHpa, the enzymes are octameric whatever the conditions. Thus, in the apo state, a single octameric species was detected for IMPDHlpp (Fig. 2B), IMPDHnm and IMPDHpa with a frictional ratio of 1.3 or 1.4 (Table 3) compatible with an ellipse with size ratio of 3.2, 3.9 and 3.7 respectively. The dependence over concentration showed a repulsive non-ideality that might not favor further oligomerisation. Furthermore, in all the other tested conditions, IMPDHlpp, IMPDHnm and IMPDHpa exhibited sedimentation coefficient values between 14.9 and 16.6 S, corresponding to an octameric form. On the other hand, the quaternary structure of the second class (IMPDHba, IMPDHbt, IMPDHsa) oscillates between tetramers and octamers. IMPDHbt (Fig. 2A) and IMPDHsa were found to be tetrameric in the apo state at all investigated concentrations with no trace of any octameric species. Extrapolation to zero concentration gave a frictional ratio of 1.4, compatible with an ellipse with size ratio of 5.0 corresponding to a similar elongated shape for both IMPDHbt and IMPDHsa. In contrast with class I IMPDHs, the dependence over concentration showed a repulsive non-ideality that may favor further oligomerisation. In the case of IMPDHkp, several peaks were detected, which could correspond to tetrameric species (8.8 S) and higher oligomeric forms, multiples of tetramers, under equilibrium (Fig. 2C), and they were not further analyzed. In the presence of IMP, class II IMPDHs remained tetrameric. Remarkably, they changed into octameric species in the presence of MgATP (without or with IMP). Moreover, in the presence of NAD, IMPDHbt and IMPDHba were predominantly octameric, whereas two forms (tetrameric and octameric species) were clearly observed for IMPDHsa. Similar results have been observed for the tetrameric species of IMPDHkp, where NAD triggers octamerisation and the octamer species remain more stable in the presence of MgATP. However, significant amount of higher order species were detected. One hypothesis to explain this behavior could be that IMPDHkp is able to form fibers as described for human IMPDH1 [16]. These data showed that NAD may favor octamerisation of these class II IMPDHs, even if the octameric species is less stable than in the presence of MgATP. It has been established previously that both substrates bind randomly to IMPDH [9]. However, our results clearly show that only NAD triggers IMPDH octamerization. This octamerisation process would have to be replaced into the context of the complex kinetic mechanism and conformational transitions of the IMPDH reaction that have been described so far [9].

**Table 3:** Oligomeric state of seven bacterial IMPDHs in their apo form determined by analytical ultracentrifugation.

	IMPDHlpp	IMPDHnm	IMPDHpa^‡^	IMPDHbt	IMPDHba	IMPDHkp	IMPDHsa
S^0^_20, W_ (S)	16.6 ± 0.1	16.3 ± 0.1	14.5 ± 0.2	9.4 ± 0.1	9.0 ± 0.1	8.8 ± 0.1*	9.3 ± 0.1
f/f_0_	1.3	1.3	1.4	1.4	1.3	1.3*	1.4
Oligomeric state	8	8	8	4	4	4*	4

For each IMPDH, runs at four different concentrations (from 9.15 to 54.9 μM for IMPDHba, from 1.85 to 76.8 μM for IMPDHbt, 1.84 to 55.2 μM for IMPDHkp, 1.83 to 54.8 μM for IMPDHlpp, 1.84 to 55.1 μM for IMPDHnm, 1.80 to 120 μM for IMPDHpa and 1.82 to 54.5 μM for IMPDHsa) were performed and absorbance at 280 nm was measured. Hydrodynamics characteristics were extrapolated to zero concentration. Sedimentation coefficients were expressed in Svedberg unit (1S = 10^-13^s^-1^) at 20°C in water. The frictional ratio f/f_0_ is characteristic of the elongation and hydration when compared to an anhydrous sphere.

^‡^Values taken from Labesse *et al*. [16].

*Higher order species are also present, see text.

**Table 4:** Hydrodynamic characteristics of six bacterial IMPDHs at a single concentration (1mg/mL corresponding to 18.3, 18.4, 18.6, 18.5, 18.3, 18.4 and 18.2 μM for IMPDHlpp, IMPDHnm, IMPDHpa, IMPDHbt, IMPDHba, IMPDHkp and IMPDHsa, respectively) in the absence or presence of IMP (10 mM), NAD (7 mM for IMPDHsa; 3 mM for the others) and MgATP (3 mM).

			apo form	IMP	NAD	MgATP	IMP + MgATP
Class I	IMPDHlpp	S_20, W_ (S)	16.6 ± 0.6	NC*	15.9 ± 0.2	16.0 ± 0.2	16.2 ± 0.5
		f/f_0_	1.20	NC*	1.25	1.25	1.25
	IMPDHnm	S_20, W_ (S)	15.3 ± 0.1	NC*	15.3 ± 0.1	16.0 ± 0.3	15.4 ± 0.1
		f/f_0_	1.30	NC*	1.30	1.25	1.30
	IMPDHpa	S_20, W_ (S)	16.4 ± 0.5	14.9 ± 0.1	15.3 ± 0.1	14.7 ± 0.3^‡^	15.2 ± 0.3
		f/f_0_	1.20	1.35	1.30	1.35^‡^	1.30
Class II	IMPDHbt	S_20, W_ (S)	9.4 ± 0.2	9.7 ± 0.4	15.5 ± 0.3	15.9 ± 0.2	15.2 ± 0.2
		f/f_0_	1.35	1.35	1.30	1.25	1.30
	IMPDHba	S_20, W_ (S)	9.6 ± 0.2	9.6 ± 0.3	14.4 ± 0.6	14.9 ± 0.2	-
		f/f_0_	1.30	1.30	1.35	1.30	-
	IMPDHkp	S_20, W_ (S)	8.8 ± 0.1^†^	NC*	14.9 ± 0.4^†^	15.7 ± 0.3^†^	14.7 ± 0.2^†^
		f/f_0_	1.30^†^	NC*	1.30^†^	1.30^†^	1.30^†^
	IMPDHsa	S_20, W_ (S)	8.8 ± 0.1	8.9 ± 0.1	10.0 ± 0.6 and 14.0 ± 0.9	15.4 ± 0.1	15.6 ± 0.4
		f/f_0_	1.40	1.40	1.25 and 1.40	1.30	1.30

Measurements were performed by interference.

* NC: not calculated as higher undetermined oligomers were detected.

^‡^Values taken from Labesse *et al*. [16].

^†^Higher order species are also present, see text.

### Steady state kinetic parameters

Analysis of the purified recombinant enzymes revealed that they were all functional IMPDHs, including IMPDHab. Optimal pH and K^+^ concentration were determined for each enzyme. All IMPDHs studied herein have an optimal pH around 8 (but IMPDHsa and IMPDHlpp with an optimal pH above 9) and required the presence of K^+^ cation at a minimal concentration of 20 to 150 mM (see Table 2).

**Fig 3:**
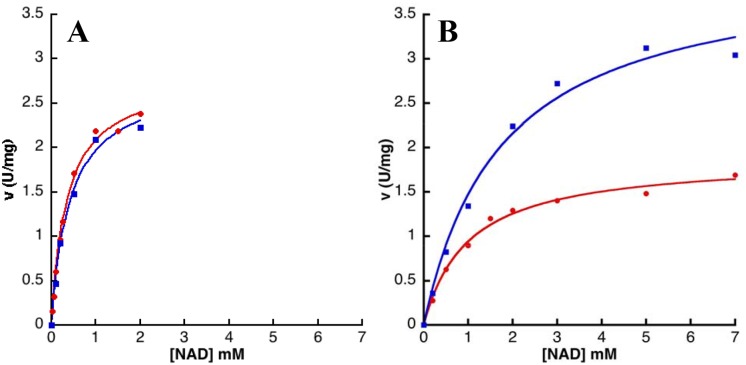
IMPDHbt (A) and IMPDHlpp (B) activities versus NAD concentration. Enzyme activity was determined at a fixed concentration of IMP (1 mM for IMPDHbt and 2 mM for IMPDHlpp), and in the absence (red curve) or in the presence (blue curve) of 5 mM MgATP. The curves correspond to the fit of the experimental data to the Michaelis-Menten equation and the calculated parameters are displayed in Table 4.

**Fig 4:**
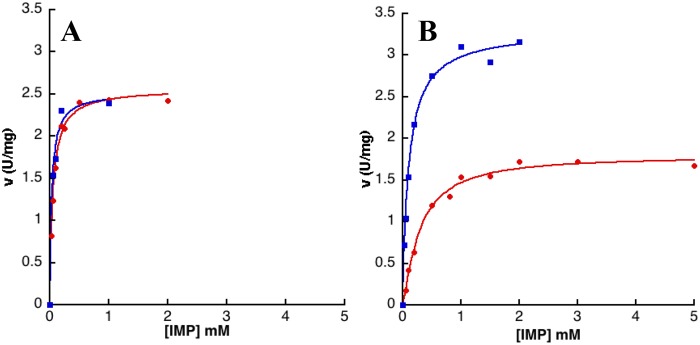
IMPDHbt (A) and IMPDHlpp (B) activity versus IMP concentration. Enzyme activity was determined at a fixed concentration of NAD (2 mM for IMPDHbt and 6 mM for IMPDHlpp), and in the absence (red curve) or in the presence (blue curve) of 5 mM MgATP. The curves correspond to the fit of the experimental data to the Michaelis-Menten equation for IMPDHbt and Hill equation for IMPDHlpp and the calculated parameters are displayed in Table 5.

Plots of initial velocity versus NAD concentration were hyperbolic, consistent with Michaelis-Menten kinetics (Fig. 3 and Table 5). The K_m_^NAD^ values vary from 139 ± 14 to 2113 ± 285 μM. A slight inhibition by excess of NAD was only observed for IMPDHbt (Fig. 3A). Concerning the dependence of activity as a function of IMP concentration (Table 6 and Fig. 4), IMPDHbt, IMPDHba, IMPDHkp and IMPDHsa (class II IMPDHs) exhibited again Michaelis-Menten kinetics. Our kinetic analysis of IMPDHba was slightly different (lower affinity for both substrates) from the work of Makowska-Grzyska *et al*. [23]. Remarkably, the plots of activity of class I IMPDHs (IMPDHlpp, IMPDHnm and IMPDHpa) as a function of IMP concentration were sigmoidal. However the n_H_ values did not exceed 1.6 and the affinity for IMP was low (K_0,5_ around 300 μM for IMPDHlpp and IMPDHnm, and 1800 μM for IMPDHpa) compared to other bacterial and non-bacterial IMPDHs [24]. Bacterial IMPDHs were reported to bind IMP efficiently and to have a low affinity for NAD [24]. Our characterization of seven other bacterial IMPDHs indicates that the situation is not that clear-cut.

**Table 5:** Kinetic parameters of seven bacterial IMPDHs, with NAD as variable substrate. Reaction rates were determined at a constant saturating concentration of IMP (1 mM for IMPDHbt and IMPDHkp; 2 mM for IMPDHba, IMPDHsa and IMPDHlpp; 3 mM for IMPDHnm; 7 mM for IMPDHpa) and in the absence or presence of MgATP (2 mM for IMPDHnm; 3 mM for IMPDHpa; 5 mM for IMPDHlpp), and fitted according to the Michaelis-Menten equation (see under Materials and Methods).

	Enzyme	Without MgATP			With MgATP		
		V_m_ (U/mg)	K_m_ (μM)	K_i_	V_m_ (U/mg)	K_m_ (μM)	K_i_ (μM)
Class I	IMPDHlpp	1.88 ± 0.06	998 ± 96	-	4.06 ± 0.19	1762 ± 281	-
	IMPDHnm	1.62 ± 0.03	269 ± 22	-	3.48 ± 0.07	477 ± 29	-
	IMPDHpa*	2.26 ± 0.07	139 ± 14	-	9.00 ± 0.5	498 ± 44	4067 ± 550
Class II	IMPDHbt	2.82 ± 0.07	355 ± 27		2.78 ± 0.14	418 ± 60	-
	IMPDHba	6.53 ± 0.19	2209 ± 232	-	5.93 ± 0.36	1994 ± 264	-
	IMPDHkp	14.00 ± 0.10	1175 ± 103	-	13.80 ± 0.30	1122 ± 65	-
	IMPDHsa	11.10 ± 0.12	2350 ± 215	-	11.80 ± 0.50	1762 ± 230	-

*Values for IMPDHpa were taken from Labesse *et al*. [16].

ND: not determined.

**Table 6:** Kinetic parameters of seven bacterial IMPDHs, with IMP as variable substrate. Reaction rates were determined at a constant concentration of NAD (2 mM for IMPDHbt, IMPDHba and IMPDHpa; 3 mM for IMPDHnm; 5 mM for IMPDHkp; 6 mM for IMPDHlpp and IMPDHsa) and in the absence or the presence of MgATP (2 mM for IMPDHnm; 3 mM for IMPDHpa, IMPDHbt, IMPDHba, IMPDHkp and IMPDHsa; 5 mM for IMPDHlpp), and fitted according to the Michaelis-Menten equation (IMPDHbt, IMPDHba, IMPDHkp and IMPDHsa) or the Hill equation (IMPDHpa, IMPDHlpp and IMPDHnm) (see under Materials and Methods).

	Enzyme	Without MgATP			With MgATP		
		V_m_ (U/mg)	K_m_ or K_0,5_ (μM)	n_H_	V_m_ (U/mg)	K_0,5_ (μM)	n_H_
Class I	IMPDHlpp	1.79 ± 0.07	297 ± 30	1.24 ± 0.12	3.31 ± 0.12	106 ± 13	0.98 ± 0.13
	IMPDHnm	1.41 ± 0.03	315 ± 18	1.38 ± 0.11	2.83 ± 0.08	50 ± 5	1.04 ± 0.15
	IMPDHpa*	2.13 ± 0.07	1760 ± 109	1.55 ± 0.08	5.58 ± 0.21	36 ± 4	0.99 ± 0.12
Class II	IMPDHbt	2.56 ± 0.04	52 ± 4	-	2.52 ± 0.12	39 ± 9	-
	IMPDHba	5.04 ± 0.27	120 ± 15	-	4.76 ± 0.42	148 ± 25	-
	IMPDHkp	11.60 ± 0.90	58 ± 14	-	10.90 ± 0.40	53 ± 6	-
	IMPDHsa	10.1 ± 0.12	196 ± 8	-	11.1 ± 0.40	197 ± 21	-

*Values for IMPDHpa were taken from Labesse *et al*. [16].

**Fig 5:**
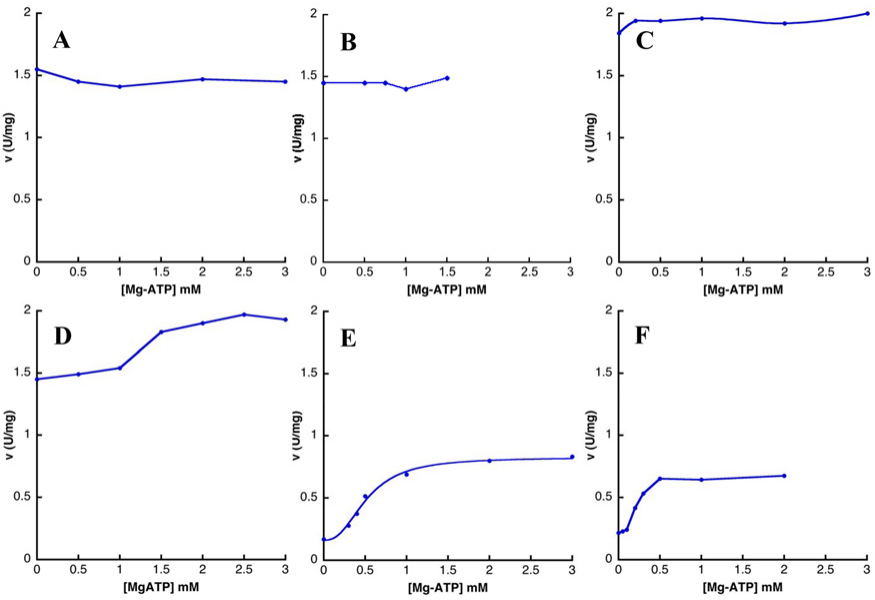
IMPDHbt (A), IMPDHba (B), IMPDHkp (C), IMPDHsa (D), IMPDHlpp (E) and IMPDHnm (F) activity versus MgATP concentration. Reaction rates were determined at constant IMP and NAD concentrations (respectively, 0.05 mM and 0.3 mM for IMPDHbt, 0.5 mM and 2 mM for IMPDHba, 0.05 mM and 0.8 mM for IMPDHkp, 0.3 mM and 1.5 mM for IMPDHsa, 0.5 mM and 0.5 mM for IMPDHlpp and IMPDHnm).

Based on our analysis of the quaternary structure described above, MgATP was evaluated on the enzymatic activity of the different IMPDHs studied herein (Fig. 5). It was found to be a positive effector for the cooperative IMPDHs (class I, namely IMPDHlpp, IMPDHnm and IMPDHpa), whereas it has no effect on the catalytic activity of IMPDHbt, IMPDHba, IMPDHkp and IMPDHsa (class II). Thus, in the presence of MgATP, the plot of activity versus the concentration of IMP became hyperbolic for IMPDHlpp (Fig. 4B), IMPDHnm and IMPDHpa. For these class I IMPDHs, both the maximal rate (Fig. 5) and the affinity for IMP (Table 6) increased significantly (a 2-fold increase of the V_m_ and a 3 to 50-fold decrease of the K_0,5_). On the other hand, the affinity for NAD decreased by a factor of 2 to 4 (Table 5). These results show that MgATP has a drastic impact on the kinetic properties of class I IMPDHs. Conversely MgATP has no significant effect on the catalytic behavior of class II IMPDHs (Figs. 3A and 4A; Tables 5 and 6).

The impact of the presence of MgATP was also examined on IMPDH thermal stability. Aliquots of 1 mg/mL protein in buffer A were heated for 10 minutes at temperatures between 30°C and 80°C, after which residual activity was determined. IMPDHlpp (representative of class I IMPDHs), and IMPDHbt (representative of class II IMPDHs) were half-inactivated at 49°C and 71°C, respectively. Addition of MgATP resulted in a significant increase of Tm values for IMPDHlpp and IMPDHbt to 67°C and 78°C, respectively (data not shown): the holo-state of these two IMPDHs is more stable than the apo-state against thermal denaturation.

### MgATP binding to class I and class II IMPDHs

The crystal structure of IMPDHpa in complex with MgATP was recently solved [16]. Sequence alignment of the eight bacterial IMPDHs studied herein (Fig. 1) showed that nearly all the residues interacting with MgATP in IMPDHpa are conserved. Thus the effector binding pockets might be preserved in both IMPDH classes, as also evidenced by the impact of MgATP on the catalytic activity of class I IMPDHs and on the quaternary structure of class II IMPDHs. In order to set up a binding assay to advance in the characterization of MgATP interaction, we have investigated fluorescence spectroscopy approaches. IMPDHpa was selected as a representative of class I IMPDHs and IMPDHbt as a representative of class II IMPDHs.

No change in the intrinsic fluorescence of IMPDHpa and IMPDHbt single tryptophan (position 261 and 260, respectively) was observed upon ATP binding.

Different ATP analogues were explored to search for positions, either on the nucleobase, the ribose or the phosphate moieties, which could be modified to introduce a fluorescent group. They were first tested using the enzymatic assay on an IMPDH which activity is sensitive to ATP concentration, namely IMPDHpa (data not shown). The only permissive position was found to be the γ-phosphate: ATP-γ-AmNS, a fluorescent ATP analogue, increased IMPDHpa reaction rate by a factor of 5.6 at non saturating concentrations of both substrates. Upon excitation at 323 nm, ATP-γ-AmNS exhibits a fluorescence emission spectrum with a maximum at 457 nm. Upon addition of IMPDHpa, there is an increase in fluorescence intensity as well as a shift of the emission spectrum to shorter wavelengths. However, no change was observed in the case of IMPDHbt, neither on the fluorescence intensity nor on the maximum emission wavelength. Moreover, measurements of fluorescence anisotropy of ATP-γ-AmNS in the absence or in the presence of IMPDHbt have not revealed any change, indicating that this ATP analogue does not bind to IMPDHbt.

**Fig 6:**
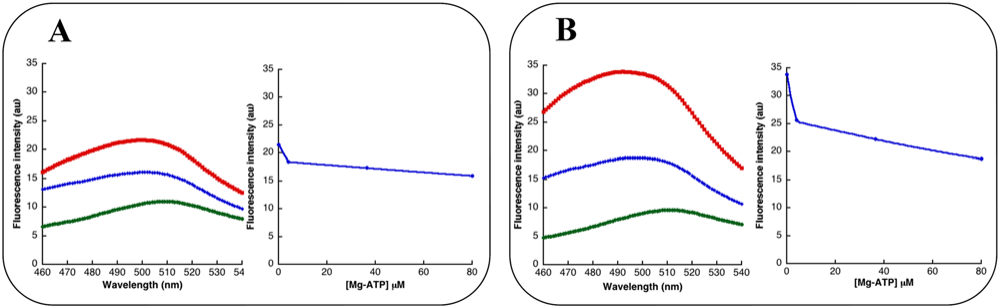
ANS binding to IMPDHpa and IMPDHbt. (**A**) Left panel: ANS fluorescence spectra alone at 10 μM (green), in the presence of 1 μM IMPDHpa without (red) or with 100 μM ATP (blue). Right panel: fluorescence intensity measured at 495 nm as a function of MgATP concentration. (**B**) The same curves for IMPDHbt.

Finally, ANS was used to highlight conformational variations upon binding of MgATP. As depicted in Fig. 6, addition of 1 μM IMPDHpa or IMPDHbt displaced the maximum emission spectrum and increased the fluorescence intensity of ANS. The plot of the fluorescence intensity of ANS measured at 495 nm as a function of ATP concentration was the same for both IMPDHs and addition of 100 μM ATP almost completely reversed the observed effects on ANS fluorescence. This suggested that the association constants of MgATP for both IMPDHs would be similar (in the μM range). Moreover, the binding of MgATP modifies the overall conformations of IMPDHpa and IMPDHbt leading to less hydrophobic regions accessible to the solvent.

## Discussion

We have characterized IMPDHs from different pathogens. On the basis of our data, we have categorized bacterial IMPDHs into two classes. Class I IMPDHs (IMPDHlpp, IMPDHnm and IMPDHpa) exhibited a cooperative kinetics for IMP with MgATP as the positive effector. For class II (IMPDHba, IMPDHbt, IMPDHkp and IMPDHsa from this study, and IMPDHs from *E*. *coli* [25,26], *Bacillus subtilis* [27,28], *Borrelia burgdorferi* [29,30], *Helicobacter pylori* [31], *Mycobacterium tuberculosis* [32,33], *Streptococcus pyogenes* as reported in the literature), the reaction rate followed the Michaelis-Menten equation for both substrates. Similarly, IMPDHs can be distinguished by their quaternary structure in the apo state into the same two classes, where class I IMPDHs are organized into octamers and class II IMPDHs are tetrameric. Whereas only IMPDHs from Gram-negative bacteria belong to class I, IMPDHs from Gram-negative and Gram-positive bacteria are found in class II. This is in contrast to bacterial UMP kinases, which represent a particular subfamily of NMP kinases, also belonging to the nucleotide metabolism. There is a clear partition of the regulation mechanism between Gram-negative and Gram-positive UMP kinases. Thus all UMP kinases from Gram-positive bacteria studied so far [34,35] exhibited cooperative kinetics for one (namely ATP) of the two substrates. On the other hand, UMP kinases from Gram-negative bacteria were not cooperative enzymes [34]. Analysis of the sequence alignment of bacterial IMPDHs (Fig. 1) did not help to identify sequence signatures associated with class I or class II. Moreover, aspartic acid 199, which was found to be a crucial residue in the IMPDHpa cooperativity [16], is absolutely conserved among all IMPDHs. Elucidation of sequence signatures for each class would need further structural and site-directed mutagenesis studies.

In eukaryotes, only class II IMPDHs would be predicted to exist as both human as well as parasitic IMPDHs are reported to be michaelien enzymes [9]. On the other hand, our recent study [16] led to a new paradigm for their quaternary structure with an octameric organization. Indeed, human IMPDH2 crystal structures analysis has revealed stable octameric assemblies. Nevertheless, it is not inconsistent with our classification proposal as all crystal structures were obtained in the presence of NAD (or inhibitors that bind in the NAD site), which, as evidenced herein, drives the equilibrium from tetrameric towards octameric species. Concerning the second human IMPDH, cryoelectron microscopy experiments of human IMPDH1 have revealed that it forms fibers in the presence of MgATP [16]. Nascent fibers formed from two types of octameric species are also present in the human IMPDH1 apo state. This behavior would resemble that of class II IMPDHkp (Fig. 2C) that can form higher order species.

**Fig 7:**
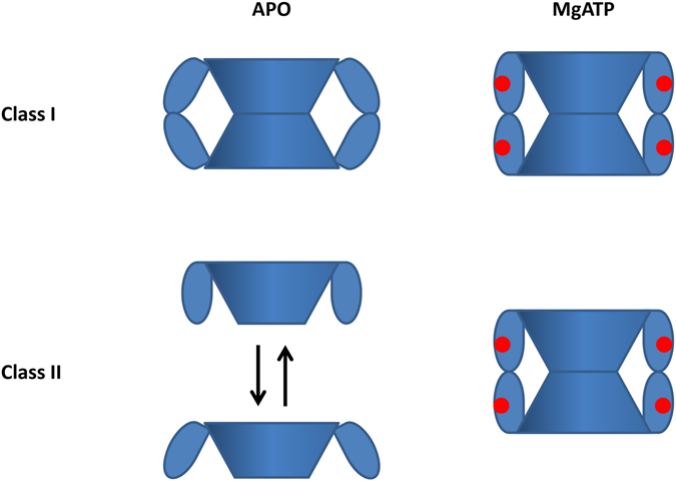
Proposed model of the modulation of the quaternary structure of class I and class II IMPDHs in the absence (APO) or in the presence of MgATP. For each tetramer, the core domain is represented as a cone and the CBS modules are on the side. MgATP are shown as red circles. Class I IMPDHs adopt an octameric architecture where the CBS modules of two tetramers interplay together in. MgATP induces a global shape rearrangement without disturbing the degree of oligomerization. Class II IMPDHs are tetrameric in the apo state. Different conformations of the tetramers would coexist and the addition of MgATP would trigger the equilibrium towards tetrameric species, which would dimerize into octamers.

CBS modules present in other proteins (DNA binding regulators, PKA associated domains, etc.) are always organized in a homodimer to form the pseudotetramer of CBS modules. These distantly related CBS tandems were recently found to bind nucleotides (mainly ADP and/or ATP) [14] at the dimerization interface. From this similarity, involvement of ATP or polynucleotides in the regulation of IMPDHs has been postulated. Whereas conflicting reports exist in the literature for the human IMPDH2 [36–39], the results described in this work demonstrated the role of MgATP in the activation of the catalytic activity of class I IMPDHs and in the modulation of the quaternary structure of class II IMPDHs and extended our previous studies [16]. Denaturation assays have also shown a thermal stabilization in the presence of MgATP. The degree of oligomerization of class II IMPDHs was modified in the presence of MgATP, as the tetramers observed in the apo state disappeared and replaced by octamers in the holo state. Considering the whole IMPDH macromolecular assembly as an ellipse, the size ratio decrease from 5.0 for the tetramers to 3.2–3.9 for the octamers: this indicated that the formation of the octamer in the presence of MgATP induce some structural rearrangements as simple piling of the two tetramers to form the octamer would have led to a two-fold decrease of the size ratio. Our hypothesis (Fig. 7) is that the CBS modules rearrange upon MgATP binding (as previously shown for a representative of class I IMPDHs, namely IMPDHpa [16]) thus favoring the octamer formation. The existence of alternative oligomeric states for the class II IMPDHs, evidenced in the absence or the presence of the substrates or MgATP might be a morpheein hallmark [6]. In the tetrameric form, the CBS modules would not be constrained and might arrange in different conformations, MgATP binding would lead to conformational changes and would drive the equilibrium of the different quaternary assemblies to tetrameric species that could interact and form octamers (Fig. 7).

Bacterial IMPDHs have generated little interest in the past and few reports described their biochemical properties (*E*. *coli* [25,26], *B*. *anthracis* [23], *B*. *subtilis* [27,28], B. *burgdorferi* [29,30], *H*. *pylori* [31], *M*. *tuberculosis* [32,33], *P*. *aeruginosa* [16] and *S*. *pyogenes* [40]). In contrast to their bacterial counterparts, eukaryotic IMPDHs have been extensively studied and have emerged as a major target for antiviral [41], antiparasitic [42], antileukemic and immunosuppressive therapies [43,44]. Meanwhile, no antibiotic exists targeting the bacterial IMPDHs, and yet consistent evidences point towards potential of this enzyme for antibacterial application [24,45]. Our discovery of an allosteric regulation of the catalytic activity of three bacterial IMPDHs (class I) opens the way for the development of allosteric inhibitors, targeting a much less conserved domain (i.e. the CBS modules) than the catalytic sites (i.e. IMP and NAD binding sites). These compounds could also be valuable for other bacterial IMPDHs, which catalytic activity is not dependent on MgATP (class II). In this case, they would modulate the quaternary structure and eventually functions yet to be discovered, and thus might have also consequences on the growth or on the fitness of these bacteria.
