# Correction: Two Classes of Bacterial IMPDHs according to Their Quaternary Structures and Catalytic Properties

**DOI:** 10.1371/journal.pone.0125884

**Published:** 2015-04-29

**Authors:** Thomas Alexandre, Bertrand Raynal, Hélène Munier-Lehmann

The second author’s name is spelled incorrectly. The correct name is: Bertrand Raynal. The correct citation is: Alexandre T, Raynal B, Munier-Lehmann H (2015) Two Classes of Bacterial IMPDHs according to Their Quaternary Structures and Catalytic Properties. PLoS ONE 10(2): e0116578. doi: 10.1371/journal.pone.0116578

There is an error in the Author Contributions. The correct contributions are: Conceived and designed the experiments: HML. Performed the experiments: TA BR HML. Analyzed the data: TA BR HML. Contributed reagents/materials/analysis tools: BR HML. Wrote the paper: BR HML.
